# *OAHG*: an integrated resource for annotating human genes with multi-level ontologies

**DOI:** 10.1038/srep34820

**Published:** 2016-10-05

**Authors:** Liang Cheng, Jie Sun, Wanying Xu, Lixiang Dong, Yang Hu, Meng Zhou

**Affiliations:** 1 College of Bioinformatics Science and Technology, Harbin Medical University, Harbin 150081, P. R. China; 2 School of Software, Harbin Institute of Technology, Harbin 150001, P. R. China; 3 School of Life Science and Technology, Harbin Institute of Technology, Harbin 150001, P. R. China

## Abstract

*OAHG*, an integrated resource, aims to establish a comprehensive functional annotation resource for human protein-coding genes (PCGs), miRNAs, and lncRNAs by multi-level ontologies involving Gene Ontology (GO), Disease Ontology (DO), and Human Phenotype Ontology (HPO). Many previous studies have focused on inferring putative properties and biological functions of PCGs and non-coding RNA genes from different perspectives. During the past several decades, a few of databases have been designed to annotate the functions of PCGs, miRNAs, and lncRNAs, respectively. A part of functional descriptions in these databases were mapped to standardize terminologies, such as GO, which could be helpful to do further analysis. Despite these developments, there is no comprehensive resource recording the function of these three important types of genes. The current version of *OAHG*, release 1.0 (Jun 2016), integrates three ontologies involving GO, DO, and HPO, six gene functional databases and two interaction databases. Currently, *OAHG* contains 1,434,694 entries involving 16,929 PCGs, 637 miRNAs, 193 lncRNAs, and 24,894 terms of ontologies. During the performance evaluation, *OAHG* shows the consistencies with existing gene interactions and the structure of ontology. For example, terms with more similar structure could be associated with more associated genes (Pearson correlation γ^2^ = 0.2428, p < 2.2e–16).

The functional annotation of human genes raises more and more attention, because it plays an important role in calculating functional similarity between human genes^1^,^2^, prioritizing disease-causing genes^3^,^4^, predicting the function of non-coding RNA (ncRNA) genes^5^,^6^,^7^,^8^,^9^ and so on.

Early studies focused on annotating the function of human protein-coding RNA genes (PCGs), because the function of PCGs could be identified directly. These annotations were sourced from functional descriptions of genes from literatures in PubMed, databases, or the experiment results, which could often be manually or automatically mapped to terminologies of biology or bioinformatics domain. In the functional annotation of PCGs domain, the earliest occurred and the most frequently used terminologies is Gene Ontology (GO)^10^, which involves three categories biological process (BP), molecular function (MF), and cell component (CC). These annotation results of PCGs with GO often be called GO Annotations (GOA)^11^. Further studies identified more aspects of the functions of PCGs, such as diseases, phenotypes, and so on. Especially, these diseases and phenotypes were annotated by Disease Ontology (DO)^12^, and Human Phenotype Ontology (HPO)^13^.

In comparison with PCGs, the function of ncRNA genes is difficult to be captured in the experiment. However, accumulating evidence indicated that more number of these types of human genes exists, especially microRNA genes (miRNAs) and long non-coding RNA genes (lncRNAs)^14^,^15^, which raises the urgency of identifying the function of miRNAs and lncRNAs^16^,^17^. Therefore, a large amount of studies gathered in exploring potential function of miRNAs and lncRNAs from multiple aspects, such as associations between miRNAs and diseases^9^,^18^, associations between lncRNAs and diseases^19^,^20^,^21^,^22^,^23^,^24^, and so on. These functional descriptions of miRNAs and lncRNAs were collected in corresponding databases. The functional descriptions of part of these databases were mapped to Medical Subject Headings (MeSH)^25^, such as Human microRNA Disease Database (HMDD) v2.0^26^ and LncRNADisease^27^. However, the descriptions of the others were not mapped to any one of terminologies yet, which limited the systematic usage of these functional descriptions.

Improved knowledge indicates that both PCGs and ncRNA genes could function in multiple levels. The functional annotations of genes could help to identify novel associations between genes. Equally, this annotation could help to identify novel associations between terms. However, disperse resources and part of unnormalized functional descriptions of PCGs and ncRNA genes are the obstacle to analyze comprehensively and effectively. Therefore, in this study, we designed an integrated framework to annotate human genes with multi-level ontologies. The integrated resource is called *OAHG*, which is freely accessible at http://bio-annotation.cn/OAHG/ or http://123.59.132.21/OAHG/.

## Results

### Statistic result of *OAHG*

**Figure 1:**
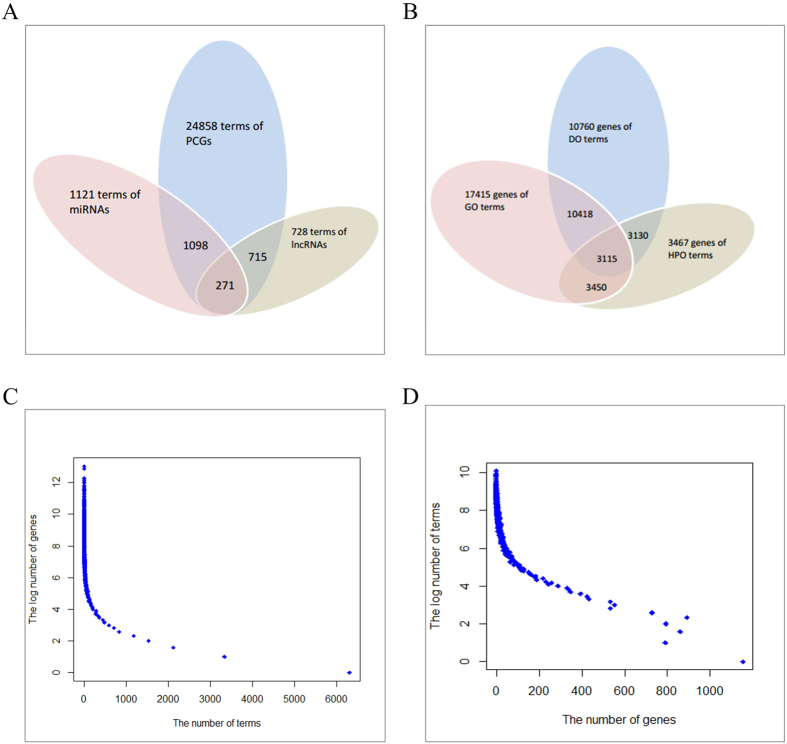
Statistical result of *OAHG*. (**A**) The distribution of terms in PCGs, miRNAs, and lncRNAs. (**B**) The distribution of genes in GO, DO, and HPO. (**C**) The number of genes associated with individual terms. (**D**) The number of terms associated with individual genes.

Figure 1A shows the number of terms associated with PCGs, miRNAs, and lncRNAs, respectively. As shown in the Fig. 1A, PCGs could be associated with the greatest number of terms (24,858 terms). In contrast, lncRNAs could be associated with the minimum number of terms (728 terms), 715 terms of which could be also associated with miRNAs and PCGs. The number of genes associated with GO, DO, and HPO, is shown in Fig. 1B. Both the number of genes associated with GO and the number of genes associated with DO are larger than 10,000. In comparison, less number of genes could be associated with HPO (3,467 genes), 3,115 of which could be also associated with GO and DO.

Figure 1C demonstrates the number of genes associated with the terms archived in the *OAHG*. 6,312 terms (6312/24894–25.4%) are associated with only one gene; while 13,124 terms (13124/24894–52.7%) are associated with more than three genes. The most prevalent GO term is ‘protein binding (GO:0005515)’, which is associated with 8,459 genes (8459/17759–47.6%). And the most prevalent DO term is ‘cancer (DOID:162)’, which is associated with 3,180 genes (3180/17759–17.9%). The number of terms associated with the genes archived in the *OAHG* is shown in Fig. 1D. 1,153 genes (1153/17759–6.5%) are associated with only one term; while 14,955 genes (14955/17759–84.2%) are associated with more than three terms. The most prevalent gene is ‘TP53 (HGNC:11998)’, which is associated with 1,103 terms (1103/24894–4.4%).

### Web interface

#### Search page

*OAHG* provides a search engine for querying detailed information on each gene-term association from our integrated resource.

**Figure 2:**
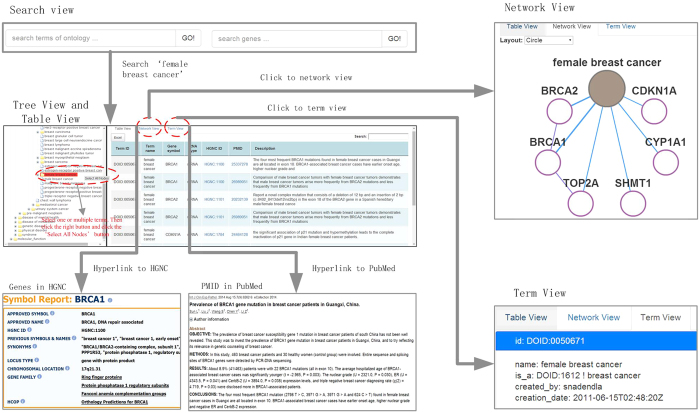
Schematic workflow of *OAHG*.

In the ‘Search View’ (Fig. 2), users can input the term name. Here, the system provides the function to automatically complete the term of ontologies and genes, which could help users to input the interested terms and genes easily. After submitting a term, system could retrieve and return the directed acyclic graph (DAG) of the term in the ontology and the associated genes of the term. The DAG of the term could be shown in the ‘Tree View’, and the gene-term association could be shown in the ‘Table View’. In the ‘Tree View’, users can hold down the ‘Control’ key, and click the left mouse button to select one or more terms. And subsequently click the right mouse button and click on the ‘Select All Nodes’ button in the web page. Then, associated genes of the selected terms could be retrieved, and these gene-term associations could be shown in the ‘Table View’. Meanwhile, two types of hyperlinks are provided in the ‘Table View’. One type of the hyperlink could link to the detailed descriptions of genes in HGNC, and the other type of the hyperlink could link to the detailed description of each gene-term association in PubMed. Besides ‘Tree View’ and ‘Table View’, *OAHG* also provides ‘Network View’ and ‘Term View’ for browsing the gene-term associations in the network visualization, and description of the selected terms, respectively. In the network page, each node represents a term or a gene, each edge between a term and a gene represents a gene-term association, and each edge between two genes represents an interaction between them.

#### Web service page

The web service page provides a client program to invoke our web services, which could return all the terms, all the genes, and gene-term associations.

### Performance evaluation of *OAHG* by gene similarity

**Figure 3:**
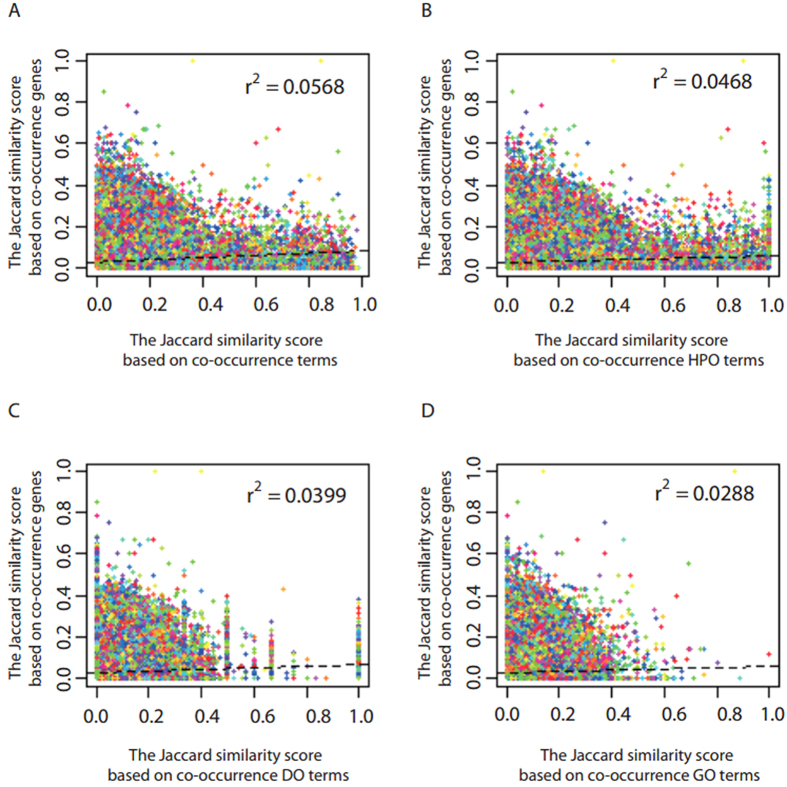
Performance evaluation of *OAHG* by gene similarity. (**A**) The distribution of the similarity of genes based on ontologies. (**B**) The distribution of the similarity of DO terms based on HPO. (**C**) The distribution of the similarity of genes based on DO. (**D**) The distribution of the similarity of genes based on GO.

Similar genes could be often interacted with collective genes. Analogously, similar genes could be often associated with collective terms. Therefore, it is expected that genes with more collective interactive genes are often associated with more collective terms. Based on this observation, we evaluated the performance of *OAHG* by comparing the consistency of the similarity of genes by their associated terms and the similarity of genes by their interactive genes. Here, gene-term associations are from the integrated resource *OAHG*, interactions between genes are from Human Protein Reference Database (HPRD)^28^ and starBase v2.0^29^, and similarity of genes is calculated by Jaccard index. As a result, similarity of genes by their associated terms was significant positively correlated with the similarity of genes by their interactive genes (Pearson correlation γ^2^ = 0.0568, p < 2.2e–16; Fig. 3A). The result suggested that genes with more collective interactive genes could be associated with more collective terms in *OAHG*.

To further verify the superiority of the integrated resource, the performance of gene-term associations based on each one of HPO, DO, and GO was evaluated by the consistency of the similarity of genes by their associated terms and the similarity of genes by their interactive genes. Results are shown in Fig. 3B–D, and the similarity of genes by their interactive genes was also significant positively correlated with the similarities of genes by their associated HPO terms (Pearson correlation γ^2^ = 0.0468, p < 2.2e–16; Fig. 3A), DO terms (Pearson correlation γ^2^ = 0.0399, p < 2.2e–16; Fig. 3A), and GO terms (Pearson correlation γ^2^ = 0.0288, p < 2.2e–16; Fig. 3A), respectively. As expected, genes with more collective interactive genes could be associated with more HPO terms, DO terms, and GO terms, respectively. Meanwhile, the performance based on each one of these three ontologies is inferior to *OAHG*. Therefore, the integrated resource could perform better in reflecting associations between genes.

### Performance evaluation of *OAHG* by term similarity

**Figure 4:**
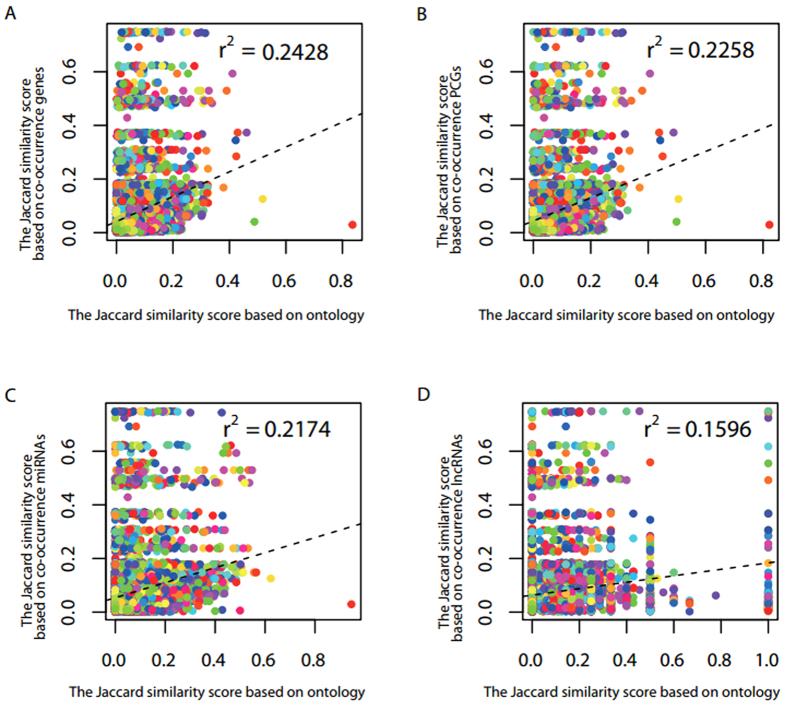
Performance evaluation of *OAHG* by term similarity. (**A**) The distribution of the similarity of DO terms based on genes. (**B**) The distribution of the similarity of DO terms based on PCGs. (**C**) The distribution of the similarity of DO terms based on miRNAs. (**D**) The distribution of the similarity of DO terms based on lncRNAs.

One way to estimate the similarity of terms of an ontology is based on the DAG of it, such as Wang’s method^30^. Another approach is based on associated genes of terms. Therefore, it is expected that terms with more similar structure based on ontology are often associated with more collective genes. Based on this observation, we needed to evaluate the performance of *OAHG* by comparing the consistency of the similarity of terms with their associated genes and the similarity of terms by the structure of an ontology. Here, the structure of DO was used for calculating the similarity between terms of DO, and associations between genes and DO terms are from the integrated resource *OAHG*. Similarity of terms based on their associated genes is calculated by Jaccard index. As a result, similarity of DO terms by their associated genes was significant positively correlated with the similarity of terms by the DAG of DO (Pearson correlation γ^2^ = 0.2428, p < 2.2e–16; Fig. 4A). The result suggested that terms with more comparable structure based on ontology could be associated with more collective DO terms in *OAHG*.

To further verify the superiority of the integrated resource, the performance of associations between genes and DO terms based on each one of PCGs, miRNAs, lncRNAs was evaluated by the consistency of the similarity of terms by their associated genes and the similarity of terms by the DAG of terms. Results are shown in Fig. 4B–D, and the similarity of terms by the DAG of DO was also significant positively correlated with the similarities of DO terms by their associated PCGs (Pearson correlation γ^2^ = 0.2258, p < 2.2e–16; Fig. 4B), miRNAs (Pearson correlation γ^2^ = 0.2174, p < 2.2e–16; Fig. 4C), and lncRNAs (Pearson correlation γ^2^ = 0.1596, p < 2.2e–16; Fig. 4D), respectively. As expected, terms with more similar structure in DO could be associated with more PCGs, miRNAs, and lncRNAs, respectively. Meanwhile, the performance based on each one type of these three genes is inferior to *OAHG*. Therefore, the integrated resource could perform better in reflecting associations between terms.

## Discussion

In the previous studies, ontologies have been proved to be very suitable for annotating the function of human genes. To gather the disperse annotation resources of human genes, we presented a framework for annotating human genes with multi-level ontologies. Then, an integrated resource called *OAHG* for annotating human genes was established, which is freely accessible at http://bio-annotation.cn/OAHG/ or  http://123.59.132.21/OAHG/. With the growing number of the identified function of human genes, more annotation resources need to be integrated. Fortunately, the framework could be extended easily to integrate more annotations of genes as required.

Statistical results in Fig. 1 (see ‘Statistic result of *OAHG*’ section) show that more annotations of PCGs than that of ncRNA genes were documented in the *OAHG*. This may be caused by that more approved PCGs were identified currently and the function of PCGs could be identified more easily than that of ncRNA genes. Meanwhile, GO is the most popular and the earliest ontology in the biological domain. Correspondingly, the greatest number of functions could be annotated by GO.

The performance of *OAHG* was validated well. And the results (see ‘Performance evaluation of *OAHG* by gene similarity’ section and ‘Performance evaluation of *OAHG* by term similarity’ section) show that the gene-term associations in *OAHG* could reflect the similarity of genes based on their associated terms and indicate the similarity of terms of an ontology based on their associated genes. Therefore, *OAHG* could be useful for identifying novel associations between genes and terms of an ontology. In comparison with functional annotations of single type of genes of PCGs, miRNAs, and lncRNAs (Fig. 4), and in comparison with functional annotations by single ontology of GO, DO, and HPO (Fig. 3), the performance of *OAHG* was always superior. These results demonstrated that the integrated resource is more comprehensive and effective.

Currently, lots of methods have been designed for prioritizing disease-related genes^31^,^32^,^33^,^34^, predicting the function of microRNAs^17^, calculating similarity between genes^35^, and etc. Obviously, sufficient knowledge of the functions of genes could be helpful for the accuracy and comprehensiveness of these methods. Fortunately, *OAHG* integrated large amount of existing functional annotations of genes. Based on the data in the *OAHG*, a multi-layer network could be even constructed, which helps for prediction of the functions of genes.

A system for querying detailed information on each gene-term association in the *OAHG* was implemented (see ‘Web interface’ section). Furthermore, gene-term associations and interactions between genes integrated into the *OAHG* could also be displayed in the ‘Network View’ (Fig. 2). The associations between multiple terms of ontologies in the network could reflect the relationships among genes and terms. Especially, terms across ontologies could also be related based on their collective associated genes, while this type of relationship is not easy to be identified based on the DAG of the ontology. In addition, web services provided by the *OAHG* were another advantage for the batch processing of data in the system locally.

## Materials and Methods

### Data Collection

#### Ontologies

Multi-level ontologies involving GO^10^, DO^12^, and HPO^13^,^36^ were downloaded in Jun, 2016 (Table 1), which provided manually curated hierarchy relationships between normalized terms. Currently, a total of 9,878 BP terms, 3,705 MF terms, and 1,549 CC terms of GO were annotated to genes in *OAHG*. In addition, 2,604 disease terms, and 7,158 phenotype terms of DO, and HPO, respectively, were also annotated to genes in *OAHG*.

#### Annotation datasets

Annotation datasets were from GOA^11^, HPO Annotation (HPOA)^37^, HMDD v2.0^26^, Gene Reference into Function (GeneRIF)^38^, LncRNADisease^27^, and lncRNAdb^39^. Among these annotation datasets, GOA and HPOA have been manually curated for annotating genes with GO, and HPO by the previous studies^11^,^37^, respectively, which could be integrated to *OAHG* directly. In comparison, diseases in HMDD, LncRNADisease, and functions of lncRNAs in lncRNAdb were manually mapped to DO, and GO, respectively. And terms in GeneRIF were annotated by Open Biomedical Annotator (OBA)^40^, which serves as a web service that annotates datasets with biomedical ontology concepts, to DO and GO. After mapping to gene symbols of HUGO Gene Nomenclature Committee (HGNC), 1,434,694 entries between 16,929 PCGs, 637 miRNAs, 193 lncRNAs and 24,894 terms of ontologies, were integrated into *OAHG*.

#### Interaction datasets

The interaction data sets were downloaded from starBase v2.0 database^29^ (Table 1), which provided experimentally confirmed mRNA-lncRNA, miRNA-mRNA, and miRNA-lncRNA interactions based on large scale CLIP-Seq data. After mapping to the gene symbols of HGNC, a total of 1,586 mRNA-lncRNA interactions between 20 mRNAs and 659 lncRNAs, 371,741 miRNA-mRNA interactions between 330 miRNAs and 11,410 mRNAs, and 2,525 interactions between 267 miRNAs and 216 lncRNAs were incorporated into *OAHG*.

The mRNA-mRNA interaction dataset was downloaded from HPRD^28^, which is a manually curated database for experimentally derived information about the human protein-protein interactions. After disposing duplicate interactions and mapping to the gene symbols of HGNC, 34,984 interactions between 8,962 mRNAs were integrated into *OAHG*.

### The framework of annotating human genes with multi-level ontologies

**Figure 5:**
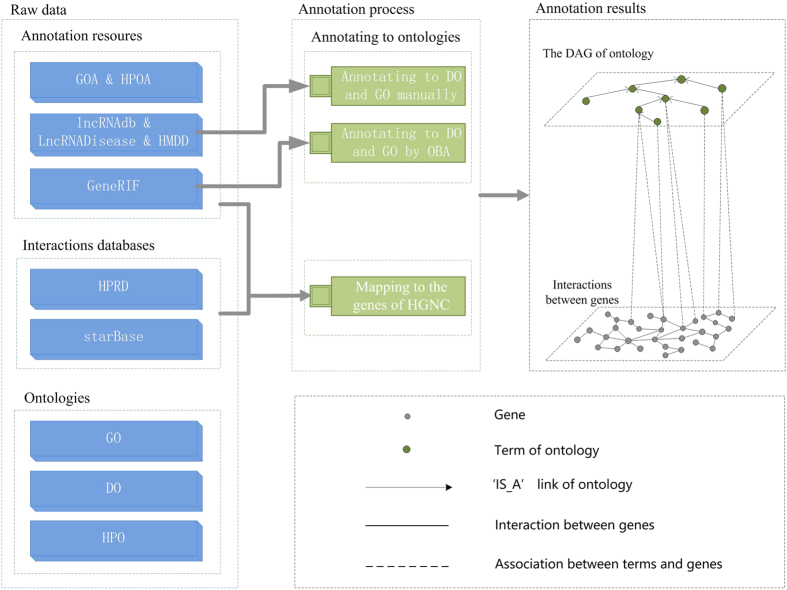
**A** framework of annotating human genes with multi-level ontologies. HPOA represents HPO Annotation. DAG represents the directed acyclic graph of ontology.

The framework of annotating human genes is illustrated in Fig. 5. Terms in GOA and HPOA have been manually annotated to DO, and HPO^11^,^37^, respectively. Therefore, both of these two resources don’t require further annotation. In comparison, terms in lncRNAdb, LncRNADisease, HMDD were manually annotated to GO and DO by ourselves. In addition, each GeneRIF was annotated to GO and DO by OBA according to the previous study^41^. The annotation results could reflect the associations between genes by their co-occurrence terms. Meanwhile, the associations between genes could also be reflected by their co-occurrence genes. Therefore, existing gene interaction databases were also integrated to reflect associations between genes from diverse views and to validate our annotation results. In addition, in order to further improve the consistency of the integrated resource, all the genes in interaction databases and annotation resources were mapped to genes of HGNC.

### Implementation

**Figure 6:**
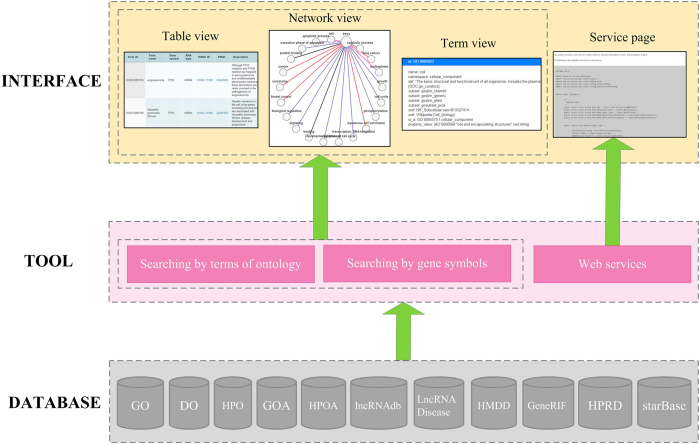
System overview of *OAHG*.

*OAHG* has been implemented on a JavaEE framework and run on the web server (2-core (2.26 GHz) processors) of UCloud^42^. The three-layer architecture involving DATABASE, TOOL, and INTERFACE layer is illustrated in Fig. 6. The detailed description of the architecture is as following.

1. DATABASE layer. This layer stores the integrated resource *OAHG*, which includes three ontologies (GO, DO, and HPO), six annotation datasets (GOA, HPOA, lncRNAdb, LncRNADisease, HMDD, and GeneRIF), and two interaction databases (HPRD, and starBase).
2. TOOL layer. Two ways have been provided for querying DATABASE layer. One way is to search by terms of ontologies, and the other way is to search by gene symbols. In addition, web services for querying annotations were also implemented, which support that *OAHG* could be accessed by the batch query.
3. INTERFACE layer. Web pages are provided for viewing annotation results, descriptions of the terms, and network visualization of associations among terms and genes. In addition, an example for invoking web services was also indicated on the web.

## Additional Information

**How to cite this article**: Cheng, L. *et al*. *OAHG*: an integrated resource for annotating human genes with multi-level ontologies. *Sci. Rep.* **6**, 34820; doi: 10.1038/srep34820 (2016).

## Figures and Tables

**Table 1:** Data sources integrated by *OAHG*.

Data source	Web sites for downloading
GO & GOA	http://geneontology.org/
HPO & HPOA	http://human-phenotype-ontology.github.io/
HMDD v2.0	http://210.73.221.6/hmdd
GeneRIF	http://www.ncbi.nlm.nih.gov/gene/about-generif
LncRNADisease	http://www.cuilab.cn/lncrnadisease
lncRNAdb	http://www.lncrnadb.org/
HPRD	http://www.hprd.org/
starBase v2.0	http://starbase.sysu.edu.cn/
DO	http://disease-ontology.org/

GOA is GO annotation, which records the association between GO terms and genes. HPOA represents HPO Annotation, which records the association between phenotypes and genes.
